# Long-Term Alterations in Motor Skills, Neurogenesis and Astrocyte Numbers following Transient Cerebral Ischemia in Mice

**DOI:** 10.3390/medicina60040658

**Published:** 2024-04-19

**Authors:** Vladimirs Pilipenko, Jolanta Upite, Beatrise Luize Revina, Baiba Jansone

**Affiliations:** Department of Pharmacology, Faculty of Medicine, University of Latvia, Raina Blvd. 19, LV-1586 Riga, Latvia; jolanta.upite@lu.lv (J.U.); beatrise_luize.revina@lu.lv (B.L.R.)

**Keywords:** angiogenesis, neurogenesis, focal ischemia, animal model, stroke recovery

## Abstract

*Background and Objectives*. Neurogenesis is an integral process in post-stroke recovery, involving the recruitment of proliferating neuroblasts from neurogenic niches of the mammal brain. However, the role of neurogenesis in the long-term restoration following ischemic stroke is fragmented. Post-stroke motor dysfunction includes challenges in the proper, coordinated use of hands and is present in roughly two-thirds of human patients. In this study, we investigated chronic behavioral and biochemical alterations after transient cerebral ischemia in adult male mice. *Materials and Methods*: Twelve-week-old C57BL/6N male mice were used, and fMCAo lasting 60 min was induced. At multiple timepoints after fMCAo induction, a single pellet reaching task was performed. Six months after the procedure, we immunohistochemically determined the number of proliferating neuroblasts (BrdU and DCX-positive) and the number of differentiated astrocytes (GFAP-positive) in both brain hemispheres. *Results*: The reaching ability of fMCAo mice was impaired from one month to six months after the induction of ischemia. Neuroblast proliferation was increased in the ipsilateral SVZ, whereas GFAP+ cell count was elevated in the hippocampal DG of both hemispheres of the fMCAo group mice. *Conclusions*: Our current report demonstrates the long-term effects of transient cerebral ischemia on mice functional parameters and neurogenesis progression. Our data demonstrate that transient cerebral ischemia promotes a long-lasting regenerative response in the ipsilateral brain hemisphere, specifically in the neurogenic SVZ and DG regions.

## 1. Introduction

Transient ischemic stroke stands as a significant contributor to global morbidity and mortality, causing over five million human deaths annually [1]. The etiology of ischemic stroke involves the blockade of blood vessels in the cerebrum and inadequate blood flow in the brain, leading to rapid cell lysis and apoptosis in the affected area, thus forming the ischemic core [2,3]. This area is formed within the initial hours after ischemia and underscores the irreversibility of brain tissue damage. Conversely, tissues surrounding the core remain salvageable. In the subacute phase, spanning up to six months post-ischemic stroke, neural repair processes come into play. This phase sees an enhancement of neurogenesis, gliogenesis and angiogenesis [4]. In the early days following an ischemic stroke, neurogenesis becomes activated to facilitate the swift recovery of cerebral tissues [5,6]. During this period, new neurons arise from neural stem cells, expressing doublecortin (DCX), a specific microtubule-associated protein, during proliferation [7]. Subsequently, these newly formed neurons start expressing NeuN, a marker of differentiated neurons [8]. Neurogenesis has been established to occur at two neurogenic sites in the brain: the subventricular zone (SVZ) located in the lateral ventricles and the subgranular zone located in the hippocampal dentate gyrus (DG) [9]. The occurrence of neurogenesis post-ischemic stroke is evident in both human [10] and rodent [11] brains, suggesting potential therapeutic interventions could enhance these processes for treating ischemic stroke clinically [6]. Neuroblast cells originating from neurogenic niches migrate to the origin site of ischemic injury, maturing into neuronal cells; however, only a small fraction of these neurons survives [12].

As shown by clinical data, approximately 75% of stroke patients are diagnosed with upper limb impairment, such as hemiparesis affecting the limb on the opposite side of the body [13,14]. This impairment significantly restricts functional independence, social engagement and the ability to resume work activities [15]. Moreover, even three months post an ischemic stroke, 50% of patients have not achieved complete recovery [16]. Additionally, 25% of survivors require professional care to manage their daily lives. Key characteristics of post-stroke motor dysfunction include challenges in skilled hand usage [17], leading to compensatory behaviors like increased trunk movement and greater reliance on the unaffected hand to compensate for hand function deficits [18].

Translating therapies for ischemic stroke from animal models to human patients has proven to be a formidable challenge [19]. Despite numerous efforts to enhance stroke research, including the creation of neurobehavioral test batteries specifically applicable to stroke models [20], Rossel and colleagues [21] noted that over 20 behavioral tests have been validated in animal stroke models. Primary testing in rodent stroke models encompasses motor, sensory and cognitive assessments, which include the corner test, cylinder test, open field test and beam walking task [22]. The evaluation of skilled reaching has been suggested as a highly effective translational behavioral test for investigating post-stroke recovery in rodents [23]. The act of food grasping with the front paws entails a highly coordinated action governed by intricate neural control mechanisms. The single pellet reaching test assesses both motor and sensory capabilities of the affected limb and is progressively employed over extended periods in research to track the recuperation process following a stroke [24,25]. Despite reports of neurogenesis taking place in the subacute stages of ischemic stroke, there are limited data on it in the non-ischemic hemisphere of the brain following stroke induction.

In this study, we induced a transient experimental ischemic stroke in healthy 3-month-old male C57BL/6N mice using a 60-minute fMCAo. At several timepoints following fMCAo, we performed a beam walking test to determine the timespan of functional behavioral deficits. Six months post fMCAo, we examined how the stroke altered the number of proliferating neuroblasts (positive for 5-bromo-2′-deoxyuridine and DCX (BrdU^+^/DCX^+^)) and differentiated astrocytes (positive for glial fibrillary acidic protein (GFAP^+^)) in the ipsilateral and contralateral neurogenic region of these mice—the subventricular zone.

## 2. Materials and Methods

### 2.1. Animals

**Figure 1:**
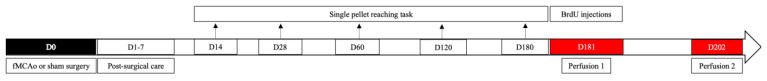
Schedule of the experiments in this study. BrdU, 5-bromo-2′-deoxyuridine; fMCAo, filament middle cerebral artery occlusion.

In this study, we used male 12-week-old C57BL/6N mice (24–27 g) that were purchased from Charles River Laboratories (Sulzfeld, Germany). Mice were randomized to a Naive, sham-operated group and an fMCAo group (*n* = 10 in each). Altogether, 30 mice were accommodated in groups of 5–6 in individually ventilated cages (GR900, Tecniplast, Buguggiate, Italy). Environmental enrichment was provided in each cage, namely autoclaved aspen wood chips (1031004, LBS-Biotech, Crawley, UK) in addition to enrichment items including a polycarbonate tunnel (K3487), crawl ball (K3329), aspen blocks (1023005) and aspen wood wool (1034005) obtained from LBS-Biotech (Reigate, UK). Each cage had preset parameters—a temperature of 25 ± 1 °C, humidity between 50 and 60%, and a 12-hour day/night cycle with lights on from 07:00 to 19:00. All mice had free access to filtered tap water and a standard pelleted chow (19.2% protein, 4.1% fat, 6.1% fiber and 5.9% ash) (1324, Altromin, Mucedola, Settimo Milanese, Italy). The experimental design of the study is provided in Figure 1.

### 2.2. Ethics Statement

The experimental design, the induction of ischemia and the use of post-operative care, as well as the implementation of humane endpoints were performed as stated in the EU Directive 2010/63/EU, ARRIVE guidelines, and local laws and policies on the protection of animals used for scientific purposes. Prior to the experiments, we received the permit (No. 100) from the Animal Ethics Committee of the Food and Veterinary Service (Riga, Latvia). We implemented all possible efforts to keep animal suffering to a minimum and to use fewer animals where possible.

### 2.3. Chemicals and Antibodies

We bought the following reagents from Sigma-Aldrich (St. Louis, MO, USA): 4′,6-diamidino-2′-phenylindole dihydrochloride (DAPI) (D9542), bovine serum albumin (A6003), Fluoromount™ compound (F4680), paraformaldehyde (PFA) (P6148), phosphate-buffered saline (PBS) (P3813), Tween^®^ 20 (P2287) and Triton X-100 (X100). Normal goat serum (NGS, 04-009-1A) was purchased from Biological Industries (Cromwell, CT, USA). BrdU (ab142567) was obtained from Abcam (Cambridge, UK). The list of antibodies used in this study can be found in Table 1.

### 2.4. Filament Middle Cerebral Artery Occlusion (fMCAo) Model

The middle cerebral artery was occluded with an intraluminal filament as described previously [26]. Briefly, we ligated the left common and external carotid arteries, then occluded the left internal carotid artery using a microvascular clip. Next, an intraluminal filament (size 7-0; 701912PK5Re suture, Doccol, Sharon, MA, USA) was inserted via a small arteriotomy in the left common carotid artery. We monitored the blood flow of mice during the procedure using a Laser Doppler flowmetry fiber (moorVMS-LDF, Moor Instruments, Axminster, UK); it was placed in a small skin incision between the left eye and the left ear. A sharp decrease (20–55% of pre-occlusion value) in blood flow was seen in the left middle cerebral artery blood supply. Reperfusion was implemented 60 min after the induction of ischemia by withdrawing the filament. Sham group mice underwent similar surgery, but the filament was withdrawn immediately after insertion to mimic the irritation of the wall of the blood vessel. Out of all the animals, two mice died from subarachnoid bleeding after fMCAo induction and were excluded from further analysis. Three days after fMCAo, we quantified the infarct volume using staining with cresyl violet, similarly as prior in [26].

### 2.5. Post-Operative Care

Post-operative care was performed as described previously [26]. Immediately after surgery, mice were s.c. injected with saline (0.5 mL) and then relocated to a recovery chamber for 2 h (with chamber temperature being 28.5 ± 0.5 °C). Overall, post-operative care was implemented for 7 days after surgery under controlled conditions. Post-operative analgesia included s.c.-injected buprenorphine (0.1 mg/kg) every 8–12 h for the first two days after surgery and i.p.-injected carprofen (5 mg/kg) every 24 hours for four days after surgery. In the post-operative period, each mouse (fMCAo and Sham) received an s.c. injection of 20% glucose saline solution (0.5 mL) and Ringer’s lactate solution (0.5 mL). Regular monitoring of signs of hypothermia and stress was performed by the caretaker of the animals.

### 2.6. Single Pellet Reaching Task

To investigate dexterous forelimb motor control in healthy mice and motor functioning deficits in mice after fMCAo, the single pellet reaching task was performed on D14, D28, D60, D120 and D180. A reaching chamber was used to determine the use of front paws in healthy mice and to assess deficits in the motor functions of the fMCAo group mice [24,25]. The chamber was made from Plexiglas and included a tray located on the outside of the wall; this tray was used to hold the pellet. The apparatus was 20 cm high, 19.5 cm long and 8 cm wide with a 1 cm wide cutout for animal limb placement. The tray for pellets was 8.3 cm long and 3.8 cm wide. Feed was taken from the mice 12 h before the test to stimulate the interest for reaching the single pellet. This pellet was put in the indention opposite to the reaching limb, thus allowing for the evaluation of unilateral brain impairments. Each mouse underwent eight-day-long training according to the protocol published by Chen et al. [25] Mice were to reach through a window and grasp and retract the pellet. The following scoring was implemented: when mice were able to retract the pellet, 1 point was given, whereas if the pellet was reached but not retracted (falling out of the paw), 0.5 points were scored. Each mice received 20 tries; the success rate was calculated from these tries using the following formula: (amount of successful reaching times/total amount of reaching tries × 100).

### 2.7. BrdU Injections

BrdU was dissolved in 0.9% NaCl and prepared in a concentration of 100 mg/mL. Each animal received two injections of 100 mg/kg BrdU. Injections were administered at approximately 07:00 and 19:00. BrdU was gradually warmed and stirred on a hotplate until it dissolved (at about 60 °C). After the solution had cooled and reached room temperature, it was loaded in a syringe (in volumes of 1 mL/kg body weight) and injected i.p. into mice. To evaluate the effects of BrdU on neuroblast proliferation and survival at two different time points, one subset of mice (*n* = 15) was perfused 12 hours after the last BrdU injection (on day 181) and processed for BrdU and DCX immunohistochemistry, and another subset (*n* = 15) was perfused 21 days after the final BrdU dose (on day 202) and processed for BrdU and GFAP immunohistochemistry.

### 2.8. Brain Tissue Preparation

On day 181 or 202 after fMCAo, mice were put into deep anesthesia by being injected with an i.p. mix containing 100 mg/kg of ketamine and 10 mg/kg of xylazine. Transcardial perfusion was then performed with saline from the fridge. Whole brains were extracted when perfusion was terminated and were put in a fixative (4% PFA) for at least 12 h. Next, the brains were transferred to a 30% sucrose in PBS for cryoprotection for 24 h. Coronal sections with a thickness of 30 μm were then cut at −26 °C ± 1 °C using a freezing microtome (CM1850, Leica Biosystems, Richmond, IL, USA). Sections were cut according to the coordinates from the mice brain atlas: from +1.18 to –2.3 mm anterior–posterior to the bregma [27]. Within each 200 μm of these coordinates, we used three random coronal sections for histochemical and immunofluorescent assays.

### 2.9. Immunofluorescence

Brain coronal sections of mice were rinsed in PBS containing 0.5% Tween® 20 (PBST). Sections then underwent antigen retrieval in 0.01 M sodium citrate (pH = 6.0) for 20 min at 95 °C. Then, non-specific binding was blocked by incubation of sections for 1 h in 10% NGS in PBST solution. Sections were then immediately transferred to trays containing primary antibodies against DCX (1:50), GFAP (1:250) or BrdU (1:4000) and remained there overnight at 4 °C. Next morning, sections were rinsed in PBST and incubated for 1 h at 37 °C with secondary antibodies: either AlexaFluor® 488-conjugated goat anti-mouse immunoglobulins (1:500) or AlexaFluor® 594-conjugated goat anti-rabbit immunoglobulins (1:500). We then stained the cell nuclei in the sections with DAPI, mounted them, allowed them to dry at room temperature and coverslipped them with the aid of Fluoromount™ aqueous medium. Cell number quantifications and optical density measurements were conducted the same day by an investigator who was unaware of the experimental groups.

### 2.10. Image Acquisition and Processing

Following coverslipping, brain sections were digitally processed using the Nikon Eclipse Ti microscopy system (Nikon Europe B.V., Amstelveen, The Netherlands). Twenty and forty× objectives were used for that, as well as three filters—FITC, DAPI and TRITC. Three sections that were stained with cresyl violet were digitized differently by being scanned with a Pannoramic MIDI II scanner (3DHISTECH Ltd., Budapest, Hungary) equipped with a 20× objective lens. Pannoramic Viewer 1.15.2 software was used to make snapshots of brain regions of interest (ROI) in the corpus striatum. To calculate immunohistochemical and histochemical staining data, we used an open-source scientific image processing software (ImageJ version 1.54f, Bethesda, MD, USA). In ImageJ, we selected an ROI of equal size for all analyzed samples. For densitometry, we calculated the mean intensity of staining and expressed it in arbitrary units. Six sections per animal were used for BrdU/DCX and GFAP quantification.

### 2.11. Statistical Analysis

Statistical analysis and graphical representation of data were performed using GraphPad Prism software (Version 7.0; GraphPad Software, Inc., San Diego, CA, USA). To test the data for normality, we used the Kolmogorov–Smirnov test. The form of data presentation is the mean value and the standard deviation (SD). Those *p*-values that were below 0.05 were considered to demonstrate statistically significant differences between the analyzed groups. For behavioral and immunohistochemical data analysis, a two-way ANOVA was implemented, followed by a post hoc test (Holm-Sidak’s). Mean values of optical density measurements and cellular quantifications were compared between the following groups:

- Sham ipsi against fMCAo ipsi group;
- Sham contra against fMCAo contra group;
- Sham ipsi against Sham contra group;
- fMCAo ipsi against fMCAo contra group.

## 3. Results

### 3.1. Impaired Reaching Ability of fMCAo Mice

The success of fMCAo induction was determined previously [26] by sacrificing three animals 24 h after the surgery; the infarct area constituted more than 50% of the ipsilateral hemisphere (Figure S1).

**Figure 2:**
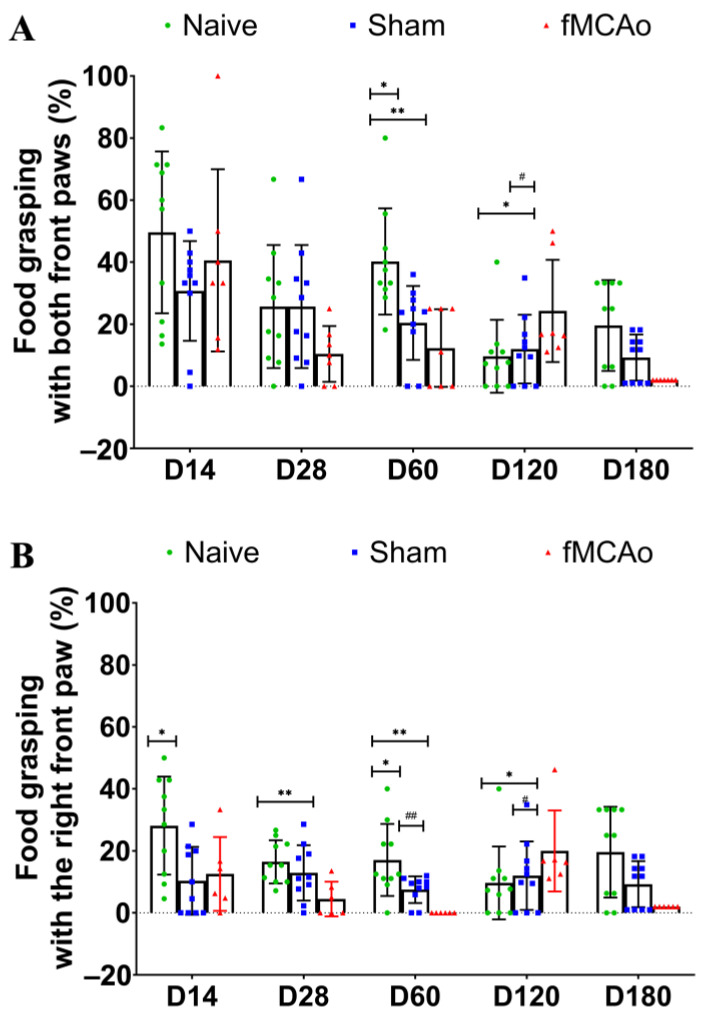
Alterations in the mice reaching ability 6 months after fMCAo. The total percentage of successfully reaching the food pellet from total tries to do so was assessed for both front paws (**A**) and for the right paw only (**B**). Data are shown as mean values (S.D.) for 7–10 mice/group. * *p* < 0.05, ** *p* < 0.01 vs. Naive group on the corresponding day; # *p* < 0.05 and ## *p* < 0.01 vs. fMCAo group on the corresponding day.

For the reaching task, we calculated the percentage of the successful amount of reaching the food from the total amount of tries. As seen in Figure 2A, the percentage of successful food grasping was significantly diminished in the fMCAo group mice. When the grasping of food with both paws was analyzed, a significant decrease in the grasping percentage was observed in the fMCAo group when compared to Naive group mice (*p* < 0.01 on D60 and *p* < 0.05 on D120) and sham-operated controls (*p* < 0.05 on D120). Additionally, sham-operated mice demonstrated significantly lower food reaching on D60 (*p* < 0.05 vs. Naive).

Next, we analyzed the percentage of reaching the food with only the right paw (Figure 2B). Here, we observed a decrease in grasping efficiency in Sham group mice in comparison to Naive group animals (*p* < 0.05 on D14 and D60). In the fMCAo group, this parameter was decreased at multiple time points following transient ischemia (*p* < 0.01 on D28 and D60, *p* < 0.05 on D180 vs. Naive). Moreover, animals in the fMCAo group grasped the food procentually less than Sham group mice on D60 (*p* < 0.01) and on D180 (*p* < 0.05).

### 3.2. Altered BrdU^+^/DCX^+^ Cell Numbers in the SVZ Region

**Figure 3:**
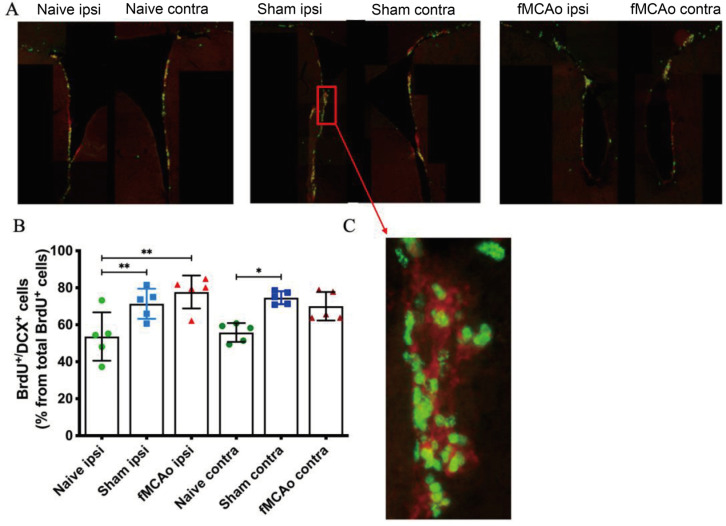
Changes in the percentage of BrdU^+^ and BrdU^+^/DCX^+^ cells in the mice SVZ 6 months after fMCAo. Representative photographs (**A**) show BrdU (green) and DCX (red) staining in the ipsilateral and contralateral mice SVZ region for all groups. Bar graphs (**B**) demonstrate quantification of BrdU^+^/DCX^+^ cell percentage from all BrdU^+^ cells. (**C**) shows a magnified (20×) image of BrdU/DCX staining. Data are shown as mean values (S.D.) for 5 mice/group. * *p* < 0.05 and ** *p* < 0.01 vs. corresponding hemisphere of Naive group.

BrdU^+^ and BrdU^+^/DCX^+^ cell counts were performed in the SVZ of the brain hemispheres of both healthy, sham-operated mice and fMCAo mice (Figure 3). A significant increase in BrdU^+^ and BrdU^+^/DCX^+^ cell percentage was observed in the ipsilateral SVZ of Sham and fMCAo group mice, compared to the same region in Naive group mice (*p* < 0.01 Sham ipsi vs. Naive ipsi and fMCAo ipsi vs. Naive ipsi). A contralateral number of BrdU^+^ and BrdU^+^/DCX^+^ cells was significantly higher in Sham (*p* < 0.05) but not fMCAo group mice compared to Naive group animals. No significant differences were observed between the ipsilateral and contralateral hemispheres within any of the groups.

### 3.3. Changes in GFAP^+^ Cells in the Hippocampal DG

**Figure 4:**
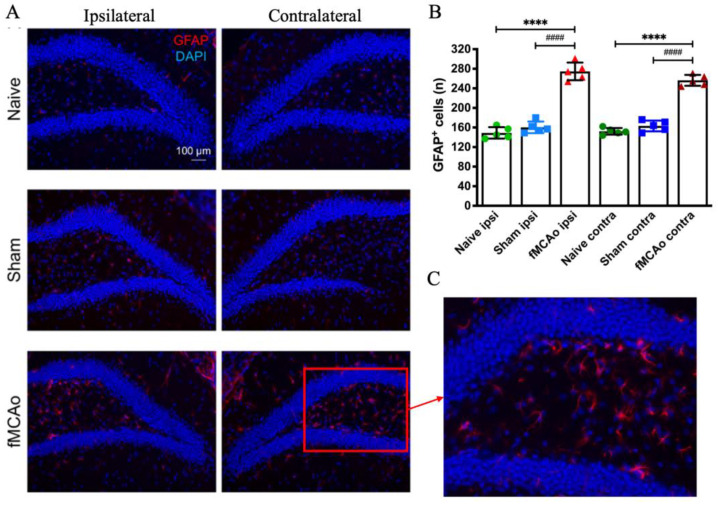
GFAP^+^ cell count in the hippocampal DG of Naive, Sham and fMCAo group mice. Representative photographs (**A**) show GFAP (red) and DAPI (blue) staining for all groups. Bar graphs (**B**) demonstrate quantification of GFAP^+^ cells. (**C**) shows a magnified (20×) image of GFAP staining. Data are shown as mean values (S.D.) for 5 mice/group. **** *p* < 0.0001 vs. fMCAo ipsi; #### *p* < 0.0001 vs. fMCAo contra.

GFAP^+^ cells were counted in the hippocampal DG of the ipsilateral and contralateral hemispheres (Figure 4). We observed a significant increase in both the ipsilateral and contralateral DG of fMCAo group mice when compared to both Naive (*p* < 0.0001) and Sham (*p* < 0.0001) group counterparts. The number of GFAP+ cells did not differ between any of the Naive and Sham group hemispheres, or between hemispheres within each of groups. 

## 4. Discussion

Neurogenesis is crucial for physiological adult brain functioning and for achieving effective restoration from neurological impairments resulting from ischemic stroke. Exploring the contributions of each cerebral hemisphere to the recovery mechanisms in the post-stroke brain holds promise for refining therapeutic strategies. Our investigation reveals that a 60-minute fMCAo triggers functional impairments, as evident by the reaching task performance. Moreover, an increase in proliferating neuroblasts in the ipsilateral SVZ and a global increase in GFAP+ cells in the hippocampal DG of ischemic model mice were observed.

The examination of skilled reaching movements across various species is extensively explored within the realms of ethology, motor control, motor learning and movement disorders. Through studying skilled motor behavior in animal models, the comprehension of the neural mechanisms underlying motor recovery following stroke has significantly progressed [28,29]. The use of the ipsilateral paw often shows impairment in stroke models involving motor cortex damage [30]. Therefore, a handedness test assessing paw preference during a food grabbing task could be valuable for identifying deficits in fine motor coordination of the forepaw in focal ischemia models. In this study, we discovered that the reaching ability of the right (contralateral) paw of fMCAo mice was diminished significantly throughout the 6-month period following the induction of ischemia.

There is little experimental data on the use of the reaching task in the chronic period after fMCAo, which opens a wide area for further research. In a 2014 study by Girard, a single pellet reaching task was used to perform the motor function assessment in male rats after 60 min fMCAo. Measurements were taken every other day, in the period ranging from the experimental day to day 25. In this study, over time, the number of successful food grain grabs increased in the control group and the fMCAo group but unevenly (resulting in the increase not being linear) [31]. In another study, the reaching task was applied to evaluate motor functions up to 13 days after ischemia; the number of successful cases increased but also not linearly [29]. In a more recent study using the rat endothelin-1 model of stroke, the reaching task was performed on experimental day 1, and days 3, 4 and 14 after stroke, and four measurements were taken individually for each rat [32]. One of the longest periods when the reaching task was implemented was 84 days (or 2.7 months) after a 90 min ischemia in rats [33]. The authors showed that the successful reaching of the food pellet was less than 50% on experimental days 28, 56 and 84.

Next, we determined changes in proliferating neuroblasts (both DCX and BrdU-positive cells) 6 months after focal ischemia. Intriguingly, their numbers were significantly increased in the ipsilateral SVZ region of the fMCAo mice brain. Our previous work, performed 2 months after fMCAo, showed diminished numbers of DCX+ cells in the fMCAo mice SVZ [26], which showcases that after an initial decline in neuroblast count, their numbers considerably rise as the process of restoration via neurogenesis takes place in the SVZ of fMCAo group mice.

We did not find similar data on altered neuroblast proliferation either in the short term or in the long term after transient ischemia. Thus far, other studies have shown that even 1 week after fMCAo, DCX^+^ cells are increased in number ipsilaterally and remain so for up to 16 weeks after experimental stroke, while it was observed that the largest BrdU^+^/DCX^+^ co-positive cell number in the brains of mice was at 8 weeks after fMCAo, while almost none were observed at 12 weeks [34]. In another study, where ischemia was induced by permanent ligation of CCA, 2 weeks after, DCX^+^ cells were found in the white matter between the ipsilateral SVZ and the ischemic area, while in the Sham group, the small DCX^+^ cells’ number was located mostly in the SVZ, where BrdU^+^ was also expressed. Nevertheless, in sham-operated animals the number of DCX^+^/BrdU^+^ cells was much lower than in those that underwent experimental ischemia [35]. In our study, a similar rise in newly formed neuroblasts was observed in the SVZ of both cerebral hemispheres in the Sham and in the fMCAo group. Our findings implicate that the chronic regenerative response following transient ischemia may involve the neurogenic niches to produce more neuroblasts. These neural precursors can then migrate to the lesioned corpus striatum and replenish the loss of cells in this region, as seen in other studies, but at an earlier timepoint of 4 months [34].

Furthermore, we detected a global rise (in fMCAo mice brain hemispheres) in GFAP^+^ cells in the hippocampal DG, another neurogenic niche. This could be attributed to the increased turnover of neuroblasts not into neurons but into astrocytes instead in this region. Reactive astrocytosis is usually observed in the first 7 days following fMCAo [36]. Other reports show an increased ipsilateral GFAP density after endothelin-1-induced ischemic stroke [32]. Increased GFAP density was also found 1 month after experimental stroke induction in the sensorimotor cortex and dorsolateral striatum of mice [37]. To the best of our knowledge, there are no reports on astrocyte number changes in the chronic stages of stroke, i.e., later than one month. Based on published reports, we can rule out that the rise of astrocytic numbers indicates the presence of a long-term inflammatory response. Most probably, neuroblasts are predominantly matured into neurons in the early post-stroke stages, whereas neuroblast maturation into astrocytes takes place in the chronic phases after ischemia.

The limitations of our study are, first of all, that we did not access other functional parameters in the reaching task, namely the number of attempts and the number of first time successful graspings [38], which would provide a more detailed picture on the sensorimotor deficits. Secondly, we do not report on neuroblast proliferation and astrocyte count in the region of the fMCAo brain that was initially the ischemic core. In fact, we performed GFAP staining in the lateral ventricles, but there were negligent GFAP^+^ cell numbers. By assessing a marker of microgliosis, Iba-1, we could be able to clarify if there is a long-lasting neuroinflammatory response in the fMCAo brain. Lastly, we only use male mice, but this is because transient fMCAo produces markedly stronger ischemic injury in them than in the female mice [39].

## 5. Conclusions

This study was performed to shed light on interhemispheric alterations in neurogenesis that take place not only between healthy and ischemic mice but also between the two brain hemispheres of a particular group (Naive, Sham or fMCAo). We found that fMCAo induces long-term functional changes that coincide with neurorestorative processes in the ipsilateral hemisphere and an increase in the astrocyte population in both fMCAo mice brain hemispheres. Further research is required to investigate if the increased astrocyte density in the hippocampal DG is associated with the proliferation of astrocytes or is a long-term neuroinflammatory response. Our data on the long-term restoration after experimental transient ischemia demonstrate enhanced neurogenesis in the SVZ and elevated astrocyte numbers in the hippocampal DG. We think that our research will gather interest for evaluating long-term changes in both brain hemispheres following an ischemic stroke, and that these observations will improve the research for novel cardiovascular agents.

## Figures and Tables

**Table 1:** List of the antibodies used in this study.

Antibody	Manufacturer	Concentration	Cat. No.	RRID
Mouse anti-BrdU	Thermo Fisher Scientific (Waltham, MA, USA)	1:4000	MA3-071	AB_10986341
Goat anti-mouse IgG2A (AlexaFluor^®^ 488-conjugated)		1:1000	A21131	AB_2535771
Rabbit anti-DCX	Abcam (Cambridge, UK)	1:50	ab207175	AB_2894710
Rabbit anti-GFAP		1:250	ab68428	AB_1209224
Goat anti-rabbit IgG (AlexaFluor^®^ 594-conjugated)		1:1000	ab150084	AB_2734147

## Data Availability

The data presented in this study are available from the corresponding author upon request. The data are not publicly available due to privacy reasons.

## Supplementary Materials

The following supporting information can be downloaded at: https://www.mdpi.com/article/10.3390/medicina60040658/s1, Figure S1. Microphotograph showing the staining of Nissl bodies in the corpus striatum of fMCAo group mice brain. Red line indicates the lesioned area. Imagewas taken using Pannoramic Midi Scanner using 2× magnification.
