# Supplementary material for: Long-Term Alterations in Motor Skills, Neurogenesis and Astrocyte Numbers following Transient Cerebral Ischemia in Mice
Source: Medicina (Kaunas). 2024 Apr 19;60(4):658. doi: 10.3390/medicina60040658 (PMC11052140; doi:10.3390/medicina60040658)
Supplement: Supplementary file 1 [file medicina-60-00658-s001.zip › medicina-2945876-supplementary.pdf]

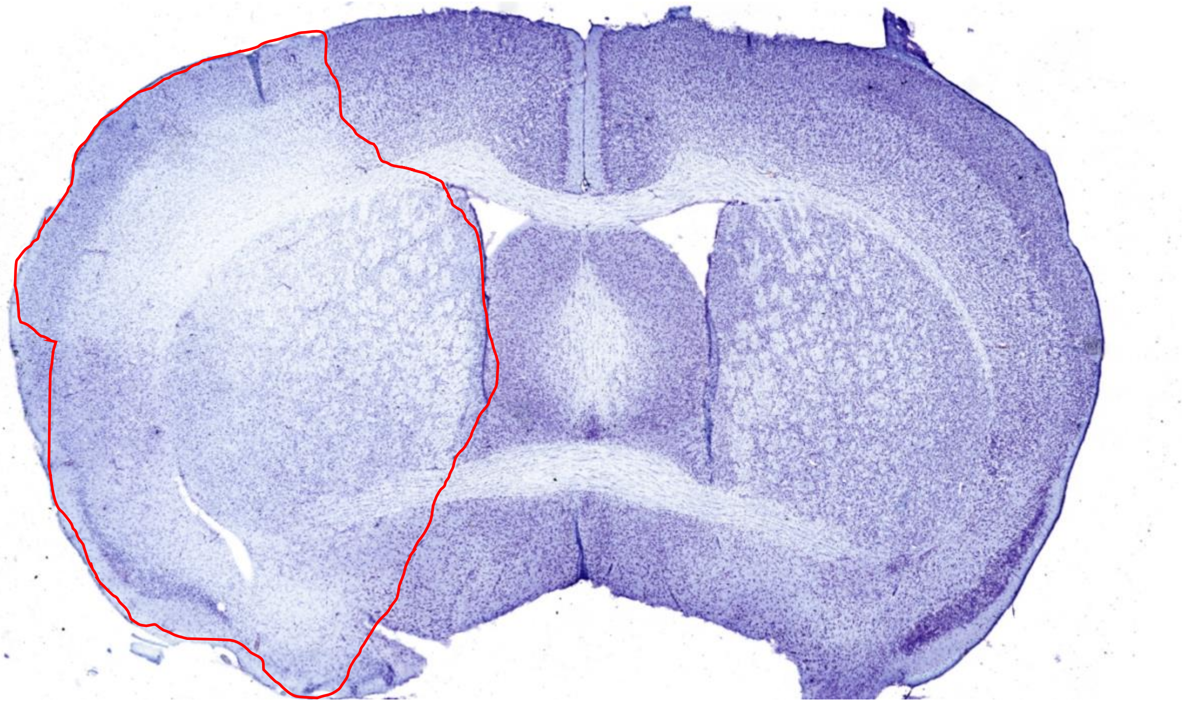

Supplemental Figure S1. **Microphotograph showing the staining of Nissl bodies in the corpus striatum of fMCAo group mice brain.** Red line indicates the lesioned area. Image was taken using Pannoramic Midi Scanner using 2X magnification.
